# Inorganic Selenium Transformation into Organic Selenium by *Monascus purpureus*

**DOI:** 10.3390/foods12183375

**Published:** 2023-09-08

**Authors:** Nan Sun, Hui Dang, Yuyao Zhang, Mengjie Yang, Wei Zhang, Yu Zhao, Haisheng Zhang, Hua Ji, Baoshan Zhang

**Affiliations:** 1 College of Food Engineering and Nutritional Science, Shaanxi Normal University, Xi’an 710119, China; 2 Research Center of Fruit and Vegetable Deep-Processing Technology, Xi’an 710119, China

**Keywords:** *Monascus purpureus*, inductively coupled plasma optical emission spectrometry (ICP-OES), sodium selenite, monacolin K

## Abstract

Selenium (Se) is a trace element that plays a crucial role in metabolism; a lack of selenium reduces the body’s resistance and immunity, as well as causes other physiological problems. In this study, we aim to identify favorable conditions for improving organic selenium production. The functional microbe *Monascus purpureus*, which is widely used in food production, was employed to optimize selenium-enriched culture conditions, and its growth mode and selenium-enriched features were investigated. Spectrophotometry, inductively coupled plasma optical emission spectrometry (ICP-OES), and HPLC (High-Performance Liquid Chromatography) were used to determine the effects of various doses of sodium selenite on the selenium content, growth, and metabolism of *M. purpureus*, as well as the conversion rate of organic selenium. The best culture parameters for selenium-rich *M. purpureus* included 7.5 mg/100 mL of selenium content in the culture medium, a pH value of 6.8, a culture temperature of 30 °C, and a rotation speed of 180 rpm. Under ideal circumstances, the mycelia had a maximum selenium concentration of approximately 239.17 mg/kg, with organic selenium accounting for 93.45%, monacoline K production reaching 70.264 mg/L, and a secondary utilization rate of external selenium of 22.99%. This study revealed a novel biological route—selenium-rich *M. purpureus* fermentation—for converting inorganic selenium into organic selenium.

## 1. Introduction

Selenium (Se) is a nonmetallic trace element that is crucial for human and animal health and plays an important physiological role in metabolism [1]. Selenium shortages reduce the body’s resistance and immunity, as well as cause other physiological problems [2,3,4]. As the main source of selenium in the human body [5], the selenium content in food depends on a variety of factors, including the environment, processing methods, and soil selenium levels where the raw materials are planted. Selenium exists in two primary forms: inorganic, such as sodium selenite and sodium selenate; and organic, such as selenoproteins and selenopolysaccharides. Organic selenium is easier to absorb and use than inorganic selenium; thus, organic selenium is essential for the production of most selenium resources [6]. Its effective activity is approximately seven-fold higher than that of inorganic selenium due to the poor bioavailability and high toxicity of inorganic selenium [7,8]. However, microorganisms can convert inorganic selenium into organic selenium with low toxicity, stable properties, and high biological activity and have the advantages of a high metabolism, conversion rate, and safety, which are important for the efficient utilization of selenium resources [9,10].

Nowadays, there are three major metabolic pathways through which bacteria interact with selenium: biosynthesis, energy transduction, and detoxification [11,12]. Proteins or extracellular polymeric substances produced by microorganisms interact with elemental selenium and act as a corona of elemental selenium nanomaterials [13]. The presence of this corona upon the formation of biogenic elemental selenium nanoparticles increases their colloidal stability, affects their interaction with metal ions, and impacts their mobility [14]. Especially with regard to that, the new method of inorganic selenium transformation into organic selenium by other microorganisms is basically a blank area of research [11]. Thus, this study aimed to identify new strategies for the production of selenium-rich products by exploring a microorganism for inorganic selenium transformation into organic selenium.

*Monascus purpureus* is a filamentous fungus belonging to the families Ascomycota, Ascomycetes, Eurotiales, and *Monascus. M. purpureus* produces several natural pigments, including monacoline K (MK), in addition to ergosterol and other secondary metabolites with beneficial health functions [15]. *M. purpureus* is widely used in the production of red koji rice wine, red koji rice, and meat and is recognized as a functional fermentation strain [16,17].

In this study, we used a production strain of *M. purpureus* that is frequently used in fermented foods and has clear functions. The liquid fermentation method of *M. purpureus* was used to carry out the biotransformation of selenium to study its growth and selenium-rich characteristics, providing a theoretical foundation and technological reference for the manufacture of functional selenium-rich foods.

## 2. Materials and Methods

### 2.1. Strains and Materials

HPLC-grade acetonitrile and phosphoric acid were purchased from Sigma-Aldrich (St. Louis, MO, USA). HNO_3_, H_2_O_2_, HCL, K_3_[Fe(CN)_6_], and other AR-grade reagents were purchased from BIOTOPPED (Beijing, China). A standard selenium solution (100 g/mL) was purchased from McLean Reagents (Shanghai, China). Potato Dextrose Broth medium (PDB) and Potato Dextrose Agar medium (PDA) were purchased from Beijing Aobox Biotechnology (Beijing, China). Sodium selenite was obtained from Hunan Donghong Biotechnology (Changsha, China). *M. purpureus* (GDMCC 3.439) was purchased from the Guangdong Provincial Microbial Strain Preservation Center (Guangzhou, China).

### 2.2. M. purpureus Activation

To get the activated strain, *M. purpureus* was inoculated on a PDA solid medium at 30 °C until red fungal colonies with evident folds and hyphae formed. Subsequently, the newly grown colonies were picked and inoculated onto fresh PDA solid media at 30 °C until the colonies became obvious [18].

Activated *M. purpureus* was cultured with 50 mL of PDB for 5 days at 30 °C and 180 rpm to obtain the seed culture medium. The seed culture solution was centrifuged at 4000 rpm for 10 min. The supernatant was discarded, and the precipitate was washed twice with 0.9% sterile physiological saline (*w/v*). The sediment was diluted with sterile physiological saline to obtain a fungal suspension at a concentration of 10^8^ CFU/mL.

### 2.3. Se-Rich Culture and Growth Monitoring of M. purpureus

Sodium selenite was added to the culture medium to alter the selenium element concentration to 0.0, 2.5, 5.0, 7.5, 10.0, and 15.0 mg/100 mL. Activated *M. purpureus* was inoculated into PDB medium and cultivated at 30 °C and 180 rpm.

Every day, the OD value and biomass of the culture solution were monitored to analyze any color changes.

Hu’s [10] method for pigment detection was used as follows: 1.0 mL of culture medium for *M. purpureus* was placed in a tube and diluted to 10 mL with a 70% C_2_H_5_OH solution. The absorption wavelength of the primary yellow pigment is 389 nm, whereas that of the main red pigment is 505 nm [19]. Thus, in the following experiments, the production of red and yellow pigments by *M. purpureus* was estimated at 389 and 505 nm, respectively.

Cell dry weight was used to express fungal biomass, and the detection method was based on that described by Khoshr [20]. The *M. purpureus* culture solution was centrifuged at 3500 rpm for 10 min and washed three times with sterile water. After drying at 80 °C to a constant weight, the precipitates were weighed. Fungal biomass was calculated using Equation (1).

(1) $$\mathrm{Fungal}\;\mathrm{biomass}=\frac{100\times (m_{2}-m_{1})}{V}$$

where m_1_ is the dead weight of the centrifuge pipe (g), m_2_ is the total mass of the centrifuge tube containing dry cells (g), and V is the measured volume of the fermentation broth (mL).

### 2.4. Comparison of Se-Rich Variations in Mycelium and Spores

Activated *M. purpureus* was added to the liquid medium and incubated for nine days under optimal culture conditions, and the cenobium floating above the liquid surface was captured. The cenobium was placed in a dish and rinsed with sterile saline, and the rinse solution was placed in an Erlenmeyer flask. After filtering the washing solution with sterile cotton and washing with sterile water 2-3 times, the fat-free cotton was placed in an oven at 65 °C and baked to a constant weight. The dried cotton was gently tapped to obtain spores. Spores (1.0 g) were accurately weighed, 10 mL of sterile saline was added, and a spore suspension was obtained by shaking. The cenobium with removed spores was baked at 65 °C to a constant weight, and the dried mycelium was obtained with a scraper. The mycelia (1.0 g) were weighed accurately, 10 mL of sterile saline was added, and a mycelium suspension was obtained by shaking. The mycelium and spore suspensions are kept at 4 °C while the selenium level is determined.

The extraction and determination of selenium were based on the methods described by Vu and Oliveira, with slight modifications [21,22]. Preparation of the Se standard curve: The selenium standard solutions were attenuated at concentrations of 0, 5, 10, 15, 20, 30, 50, 100, and 200 g/L, injected into the inductively coupled plasma optical emission spectrometry (Agilent 5800 ICP-OES, CA, USA), and the Se signal response value was determined. The *M. purpureus* culture solution (3.0 mL) was precisely transferred to the digestion tube. HNO_3_ (10.0 mL) and H_2_O_2_ (2.0 mL) were added, and the mixture was shaken and allowed to stand for 15 min. The sample solutions were processed using a Microwave Digestion System. After digestion, the solution was degassed via ultrasonication for 5 min, diluted to 25 mL with sterile water, and thoroughly mixed. Blank solutions were prepared simultaneously. Subsequently, the blank solution and sample were injected into the ICP-OES. The Se ion concentration in the digestion solution was measured using a standard curve, and the signal response values of Se and the internal standard elements were measured.

### 2.5. Impact of Culture Conditions on M. purpureus Se-Enrichment

(1) Effect of pH on Se-enrichment of *M. purpureus*

After adjusting the pH of PDB with HCL or sodium hydroxide to 5, 6, 7, 8, and 9 correspondingly, *M. purpureus* was cultured at 30 °C and 180 rpm for 15 days. This experiment explored the effects of medium pH on the growth process of *M. purpureus*, including changes in fungal biomass, yellow pigment, red pigment content, and selenium content.

(2) Effect of temperature on Se-enrichment of *M. purpureus*

Culture temperatures were set at 24, 27, 30, 33, and 36 °C. The effects of culture temperature on the Se enrichment of *M. purpureus* and its growth rule were investigated under the ideal culture conditions of Experiment 1.

(3) Effect of rotational speed on Se-enrichment of *M. purpureus*

The rotational speed was adjusted to 80, 130, 180, 230, and 280 rpm. The effects of rotational speed on the development of *M. purpureus* were investigated under the ideal culture conditions of Experiment 2.

Each of the preceding experiments included three parallel groups.

### 2.6. Design of the Response Surface (RSM) Experiment

Based on the previous single-factor studies, the Box–Behnken design was used for the selenium-enhanced growth conditions. Response surface analysis was performed to determine the optimal conditions for selenium-enriched *M. purpureus*. Table 1 lists these factors and codes.

### 2.7. Selenium Conversion Rate of Se-Rich M. purpureus

The sample solution (1.0 mL) was diluted to 100 mL using distilled water. The diluent (20 mL) was centrifuged at 5000 rpm for 30 min after standing at 4 °C for 1 h. The mixture contained 10 mL of supernatant, 12.5 mL of 6 mol/L HCl, and 2.5 mL of a 0.1 g/mL K_3_[Fe(CN)_6_] solution diluted to 50 mL with distilled water [23,24]. The blank solution and sample solution were injected into an ICP-QES, the signal response values of the measuring elements and the internal standard elements were determined, and the concentration of the element was measured in the digestion solution based on the standard curve.

The determination of selenium form refers to the method of Vacchina and Liu et al. [25,26]. The ICP-MS used was an Agilent 7500 cx (for HPLC-ICP-MS measurements). Chromatographic separations were carried out using a Model 1200 HPLC pump (Agilent, Wilmington, DE, USA) as the delivery system. The outlet of the column was directly connected to the nebulizer of the ICP-MS by means of PEEK tubing. Injections were performed using a Rheodyne valve with a 100-μL sample loop.

### 2.8. Effects of Sodium Selenite on M. purpureus Physiological Activity

A culture medium was used to examine the physiological activity of Se-rich *M. purpureus* and its secondary cultures. The monacoline K content of Se-rich *M. purpureus* was measured.

Monacoline was extracted and determined based on Liu’s [27] method. Briefly, C_2_H_5_OH (30 mL of 75%) was added to 10 mL of the sample, and the mixture was extracted by ultrasound (20 kHz) at 50 °C for 30 min. After centrifugation at 4000 rpm for 10 min, the supernatant was filtered with an ultrafiltration membrane (0.45 μm), and the volume was fixed to 10 mL. For HPLC analysis, a ZORBAX SB-C18 chromatographic column (150 mm × 4.6 mm, 5 μm) was used with a column temperature of 25 °C, flow rate of 1.0 mL/min, an injection volume of 20 L, and a UV detector wavelength of 237.0 nm. The mobile phase for the gradient elution analysis was a 75% acetonitrile solution (pH 2.5 adjusted with phosphoric acid).

### 2.9. Characteristics of the Second-Generation Strains of M. purpureus after Se-Enriched Culture

*M. purpureus* cultured for five days with Se enrichment under optimal conditions was further cultured for 15 days at 30 °C and 180 rpm in PDB medium without sodium selenite. Changes in selenium content in the mycelia and culture medium were measured. At the same time, *M. purpureus* cultured with selenium was inoculated onto cooked rice and cultured under optimal fermentation conditions for 15 days.

The rice color and coloring rates were measured. Color value determination methods refer to Embate and Bhat et al. [28,29]. The sample was crushed by a crusher and passed a 250 μm~380 μm sieve. Accurately weigh 0.2 g (accurate to 0.001 g) of the sample to be crushed and mixed evenly. The solution temperature should be allowed to return to normal after removing the sample. The solution was diluted to 100 mL with a 70% ethanol solution and thoroughly mixed. It was filtered using filter paper and collected in a stoppered colorimetric tube. The filtrate was extracted from 5.0 mL into a 50 mL volumetric flask, diluted to 50 mL with a 70% ethanol solution, and thoroughly mixed. The 70% ethanol solution was used as a reference, and the absorbance of the sample was measured at 505 nm.

### 2.10. Data Processing

Excel software was used to present the experimental data, and SPSS 22.0 (Version: IBM SPSS Statistics 25) and Design-Expert 12 (Version: 12.0.3.0 64-bit) software were used to process the data and analyze the significance of the differences. Each measurement was performed in triplicate.

## 3. Results and Discussion

### 3.1. Subsection Se-Rich Growth and Metabolism of M. purpureus

**Figure 1:**
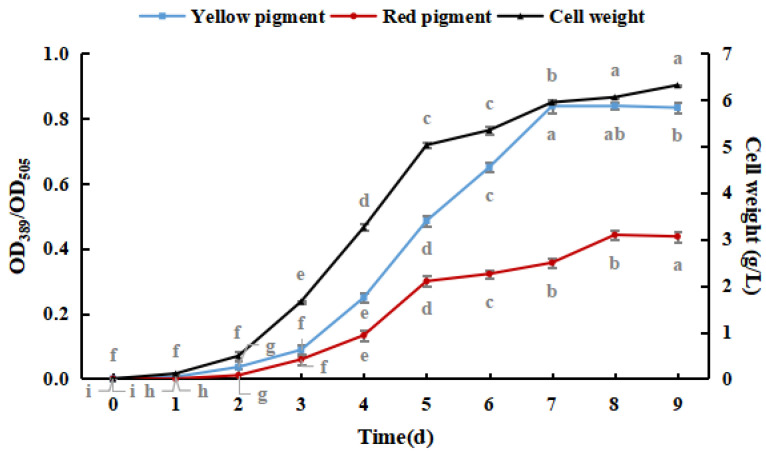
Effect of culture time on the growth of *Monascus purpureus.* (The grey lowercase letters in the graph are used for significance difference analysis).

As shown in Figure 1, *M. purpureus* pigment production and cell weight increased with culture time. Yellow pigment production and cell weight gradually stabilized after seven days, and red pigment production gradually stabilized after eight days. The production of the yellow pigment was greater than that of the red pigment during the culture process, and red pigment production peaked earlier than that of the yellow pigment. The production of pigments did not significantly decrease because shorter culture times and lower weights were not conducive to the production and accumulation of pigments [30]. Therefore, *M. purpureus* cultured for nine days was used as an experimental subject in subsequent experiments.

Sodium selenite was added to the PDB medium, and the selenium concentration was adjusted to 0.0, 2.5, 5.0, 7.5, 10.0, and 15.0 mg/100 mL. Afterward, *M. purpureus* was inoculated and cultured at 30 °C and 180 rpm for nine days.

**Figure 2:**
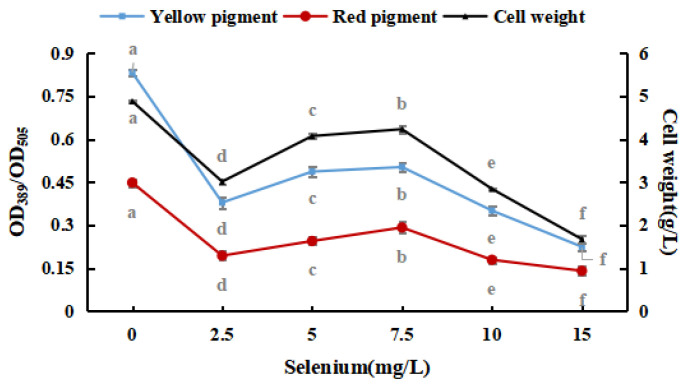
Effects of sodium selenite on weight and pigment content of *M. purpureus.* (The grey lowercase letters in the graph are used for significance difference analysis).

Figure 2 shows that *M. purpureus* had some selenium tolerance and enrichment ability; however, its growth was considerably hindered as the inorganic selenium concentration increased. Throughout the test, the sodium-selenite-treated group frequently produced red pigments faster than that of the control group. For the reason that *M. purpureus* converted and used sodium selenite, the environment was improved, and the metabolism of the strain was stimulated. The red selenium phenomenon may be caused by the microbes restoring Se^4+^ to red elemental Se, which is why the color of the liquid medium significantly increased when the selenium content in the medium was more than 10 mg/L [31,32]. The fraction of selenium converted to elemental Se increased as the selenium level in the culture medium increased, impacting the absorbance. To avoid the generation of elemental Se, liquid culture findings indicated that the selenium level in the medium should be 10 mg/L. These results revealed that the growth and metabolism of *M. purpureus* improved when the selenium concentration in the medium was 7.5 mg/100 mL. When the selenium content of the culture medium was >10 mg/L, the inhibition rate was high (91.56%).

**Figure 3:**
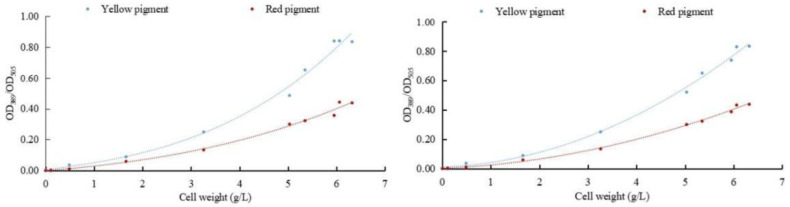
Correlation between cell weight and pigment. (The relationship between fungi and pigment metabolism is depicted on the (**left**); the relationship between selenium-rich fungi and pigment metabolism is depicted on the (**right**)).

To determine the effect of sodium selenite on the metabolism of pigments in *M. purpureus*, the relationship between the weight of *M. purpureus* and the pigment was obtained according to the data analysis (Figure 3). The regression equation between weight and red pigment was y1 = 0.0005x^3^ + 0.004x^2^ + 0.0254x − 0.0014, R^2^ = 0.9908. The regression equation between cell weight and yellow pigment was y2 = 0.0018x^3^ + 0.0045x^2^ + 0.039x + 0.0048, and R^2^ = 0.9899. The regression equations for cell weight and red pigment content after selenium addition were y1 = 0.0088x^2^ + 0.0142x + 0.0012, and R^2^ = 0.9973. The regression equation between cell weight and yellow pigment content was y2 = 0.0192x^2^ + 0.0119x + 0.0098, R^2^ = 0.9956.

The results of multiple experiments showed that the yellow and red pigment production of *M. purpureus* gradually increased with the extension of culture time without the addition of exogenous selenium and was less affected by the biomass of the cell. When exogenous selenium was added, the yields of the yellow and red pigments of *M. purpureus* increased with increasing cell weight, which was greatly affected by cell growth. When the cell biomass reached >600 mg/L, the correlation between the cell biomass and pigment was generally poor. The metabolic rate of *M. purpureus* significantly increases in the middle and late stages of growth and is limited by culture space, nutrients, and cell autolysis. When the fungal weight reached >600 mg/L, the link between pigment synthesis and fungal biomass shifted. The incubation duration for the follow-up test was set at nine days based on the growth trends and metabolic alterations of *M. purpureus*.

### 3.2. Difference in Selenium Enrichment of M. purpureus Hyphae and Spores

The selenium content in the hyphae and spores of Se-rich *M. purpureus* was determined under optimal culture conditions of 0.0, 2.5, 5.0, 7.5, 10.0, and 15.0 mg/100 mL selenium in the culture medium for nine days.

**Figure 4:**
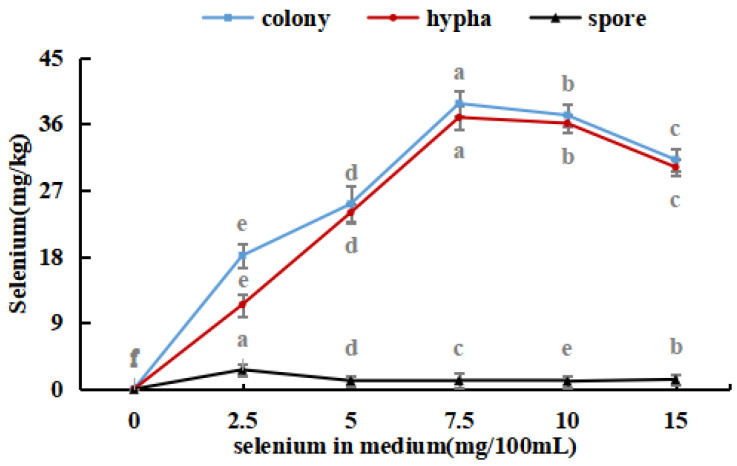
Selenium content in *M. purpureus* strains, hyphae and spores. (The grey lowercase letters in the graph are used for significance difference analysis).

As shown in Figure 4, with an increase in the selenium content of the medium, the selenium ion concentration in *M. purpureus* first increased and then decreased. The maximum selenium content in the cells was approximately 239.17 mg/kg. A significant amount of sodium selenite was transferred to the mycelium for metabolic decomposition, whereas the spores contained only a small amount of selenium. During the growth of *M. purpureus*, secondary metabolites and spores gradually accumulate, and different material structures determine the distribution of Se in the strain. Protein selenium was the main carrier of organic selenium, whereas spores had a special structure and low protein content; therefore, spores were not the main structural carriers of selenium, and their contents were low [33]. The selenium content of the strain and culture media were positively correlated, indicating that the selenium content of the culture medium was the primary determinant of *M. purpureus*’s selenium utilization rate within a given range. The findings demonstrated that the selenium enrichment effect was greatest when the selenium level was 7.5 mg/100 mL.

### 3.3. Effect of Culture Conditions on Selenium Enrichment of M. purpureus

#### 3.3.1. Effect of Nutrients on Selenium Enrichment of *M. purpureus*

After the pH of PDB was adjusted to 5, 6, 7, 8, and 9 using lactic acid or sodium hydroxide, activated *M. purpureus* was incubated at 30 °C and 180 rpm for 15 days. This experiment explored the effects of medium pH on the growth process of *M. purpureus*, including changes in fungal biomass, yellow pigment, red pigment content, and selenium content.

**Figure 5:**
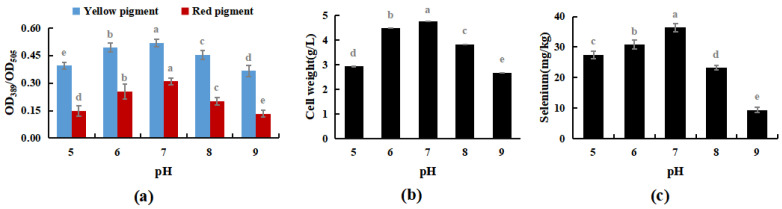
Effects of pH on selenium enrichment growth of *M. purpureus* (**a**). Effects of pH on yellow pigment and red pigment contents; (**b**). Effects of pH on the volume of bacteria; (**c**). Effects of pH on selenium content; (The grey lowercase letters in the graph are used for significance difference analysis).

The results shown in Figure 5 indicated that *M. purpureus* grew well in the presence of selenium when the pH of the culture medium was in the range of 5 to 8, and the growth impact was greatest when the pH of the culture medium was 7. Traditional fermentation mostly involves solid-state fermentation, and the raw materials for fermentation are mostly cooked or glutinous rice. Modern industrial production mostly involves liquid fermentation, and culture substrates include glucose, maltose, sucrose, glutinous rice flour, and other carbon sources. The above two production methods are mostly natural; therefore, the pH of the culture medium is mostly between 7 and 7.6 [34]. During the experiment, when the culture medium was pH 7, *M. purpureus* colonies formed early and in high numbers, whereas the size, number, and occurrence time of the colonies were approximately the same when the culture medium was pH 6 and pH 8. A culture medium at pH 7 was more appropriate for the development of *M. purpureus*, and colonies visible to the unaided eye emerged sooner and metabolized pigments according to experiments and comparisons. The growth of *M. purpureus* was significantly inhibited later in the experiment because of the limited nutrients and space in the culture medium, the excessive accumulation of waste from the growth and metabolism of *M. purpureus*, and the gradual stagnation of pigment metabolism.

#### 3.3.2. Effect of Culture Temperature on Selenium Enrichment of *M. purpureus*

The cultivation temperature was set at 26, 28, 30, 32, and 34 °C, respectively. Under the optimal cultivation conditions in Experiment Section 3.3.1, the effects of cultivation temperature on the selenium enrichment of *M. purpureus* and its growth patterns were measured.

**Figure 6:**
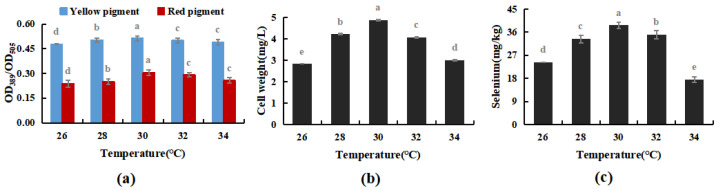
Effect of temperature on selenium-enriched growth of *M. purpureus* (**a**). Effect of temperature on yellow pigment and red pigment contents; (**b**). Influence of temperature on bacteria volume; (**c**). The effect of temperature on selenium content; (The grey lowercase letters in the graph are used for significance difference analysis).

As shown in Figure 6, within a particular range, temperature had no discernible impact on the *M. purpureus* metabolism of yellow pigment, whereas the red pigment, biomass, and selenium concentration reached their peak at 30 °C. When the temperature exceeded 32 °C, the selenium content and cell biomass decreased considerably, but the pigment content did not change significantly. The red selenium phenomenon may have had an impact on the pigment test results, whereas it’s possible that selenium invertase was the most active around 30 °C, meaning that the selenium conversion rate was high. According to Huang’s research on the *M. purpureus* life cycle, it can be hypothesized that the temperature had an impact on the process of *M. purpureus* “Ascomycete creation and generation of a tiny amount of ascomycetes” [35], increasing the biomass of *M. purpureus*, and promoting the transformation and use of selenium in the culture medium, reaching 38.7 mg/kg. Therefore, 30 °C was used as the optimal temperature for Se-rich *M. purpureus* cultivation in the following experiments.

#### 3.3.3. Effect of Oscillation Frequency on Selenium Enrichment of *M. purpureus*

**Figure 7:**
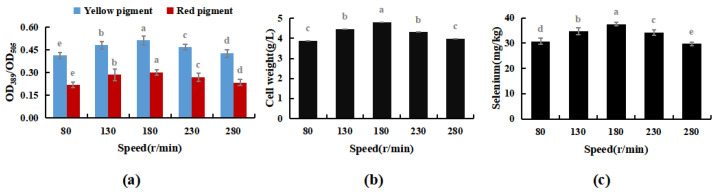
Effect of rotating speed on Se-rich growth of *M. purpureus* (**a**). Effect of rotating speed on the content of yellow and red pigment; (**b**). Effect of rotating speed on the amount of bacteria; (**c**). Effect of rotating speed on the content of selenium; (The grey lowercase letters in the graph are used for significance difference analysis).

The results shown in Figure 7 indicate *M. purpureus*’s growth did not increase with oscillation frequency. Maximal cell biomass was obtained at 180 rpm. When the rotation speed exceeded 230 rpm, the mechanical force acting on the cell increased with increasing rotation speed, and the damage to the cell was greater than that in the dominant growth environment caused by the uniform distribution of nutrients. As the test progressed, cell biomass development limited contact with dissolved oxygen and sodium selenite, which prevented *M. purpureus* from converting to sodium selenite. In conclusion, 180 rpm was shown to be the optimal rate for Se-rich cultivation of *M. purpureus*.

### 3.4. Response Surface (RSM) Test

Response surface analysis was conducted for *M. purpureus* cultivated under sodium selenite conditions in accordance with the Box–Behnken test. The response value Y (selenium content) to the coding value of each condition was calculated using the following quadratic polynomial regression equation: Y = 37.54 + 0.38A − 0.028B − 0.54C − 1.96A^2^ − 0.34B^2^ − 1.98C^2^. The results of the established model’s variance analysis and regression coefficient significance test are shown in Table 2.

As shown in Table 2, the F-value of the model determined from the test data was 131.98 (*p* < 0.0001), demonstrating the exceptional significance of the model. The developed regression equation was well-suited, with good fitting and reliability, which was beneficial to the growth of *M. purpureus*. The P of the mismatched term was 0.5505, which was not significant, and R^2^ = 0.9993. Se-rich *M. purpureus* was most significantly affected by the interaction between culture temperature and nutrients (*p* < 0.001). However, the interaction between culture temperature and rotation speed had no significant effect on the metabolism of inorganic selenium by *M. purpureus* (*p* > 0.05). The results indicated that the culture mediums adjusted to pH 6.8 at 30 °C and rotated at 180 r/min were optimal for growing *M. purpureus* in a Se-rich environment.

### 3.5. Physiological Activity of Se-Rich M. purpureus

#### 3.5.1. Selenium Conversion Rate of Se-Rich *M. purpureus*

Se-rich *M. purpureus* can produce organic selenium during the culture process by combining sodium selenite in the culture medium with biological macromolecules in the cells of *M. purpureus*. Selenium has fermentation properties and biological activity in addition to acting as an organic carrier of selenium. Organic selenium has higher bioavailability, is easier for the body to absorb, and is used for its physiological function [36]. The concentrations of organic and inorganic Se were used to assess the Se conversion rate and bioavailability in Se-rich *M. purpureus*.

**Figure 8:**
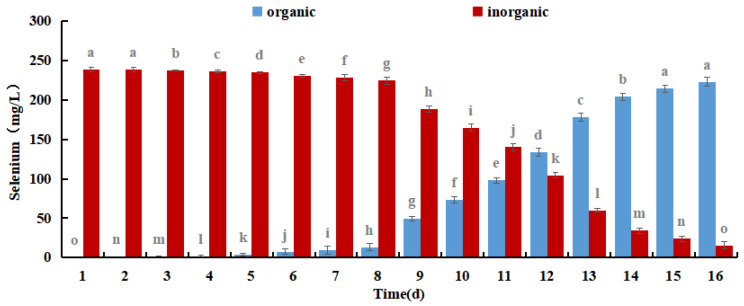
Different forms of Selenium in Se-rich *M. purpureus.* (The grey lowercase letters in the graph are used for significance difference analysis).

The results shown in Figure 8 demonstrate that the amount of organic selenium and its proportion to the overall selenium content in *M. purpureus* gradually increased over time. After 12 days of incubation, the organic selenium concentration stopped increasing because *M. purpureus* growth stabilized on day 9 and its capacity to convert inorganic selenium to organic selenium was constrained. *M. purpureus* typically enters the decay phase 15 days later. It was challenging to maintain an increase in organic selenium content as the strain degraded. According to experts, the presence and comparison of sugars and proteins affect *M. purpureus* secondary metabolism. It is anticipated that the biological activity of *M. purpureus* could be utilized in Se-rich culture procedures to increase productivity while enhancing the amount of protein and carbohydrates. These findings demonstrated that *M. purpureus* grew and functioned more effectively when the selenium content was 7.5 mg/100 mL. After 12 days of culture, the organic selenium content increased to 93.45%.

**Figure 9:**
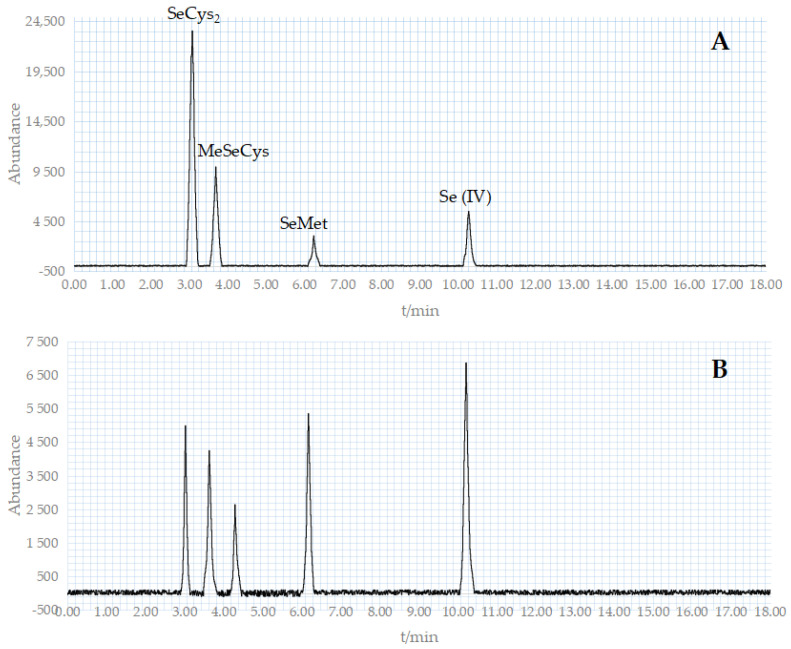
HPLC chromatogram of crude extraction as well as standard compounds. ((**A**): The HPLC chromatogram of standard compounds; (**B**): The HPLC chromatogram of crude extraction).

The benchmark for the morphological assessment in this trial was a mixture of four standard reagents: SeCys2, MeSeCys, SeMet, and Se (IV) (Figure 9A). Figure 9B depicts the appearance of five distinctive peaks in the sample determination. The holding periods of the five components were 3.02, 3.63, 4.29, 6.17, and 10.21 min, respectively. SeMet is the major form of selenium in the Se-rich *M. purpureus*, with traces of SeCys2 and MeSeCys. For the characteristic peak that appears at 4.29 min, according to literature analysis [37], the substance may be Se (VI), and further testing and verification are needed in the future.

#### 3.5.2. Se-Rich *M. purpureus* Monacoline K Output

Monacoline K (MK) is a secondary metabolite with a polyketone structure that is produced by *M. purpureus*. It has high biological activity and can effectively inhibit the synthesis of cholesterol and reduce blood lipids with few adverse effects. Therefore, the MK content of Se-rich *M. purpureus* can be used to describe the bioactive roles of the secondary metabolites [38].

**Figure 10:**
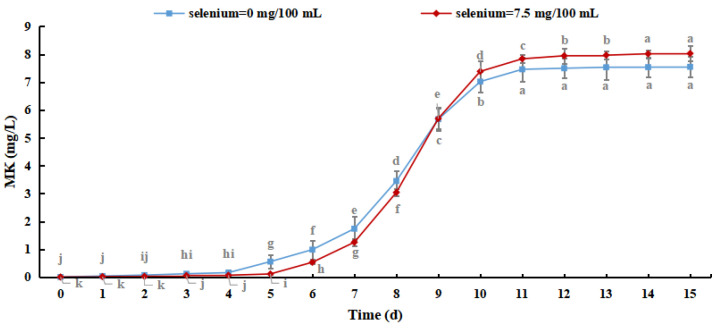
Monacoline K content in Se-rich *Monascus purpureus.* (The grey lowercase letters in the graph are used for significance difference analysis).

As shown in Figure 10, the MK content of *M. purpureus* increased with culture time. The production of MK reached 8.026 mg/L when the selenium content in the culture medium was 7.5 mg/100 mL, which was increased by 0.480 ± 0.365 mg/L compared with the control group, indicating that the selenium-enriched culture of *M. purpureus* had a promoting effect on the MK content. Although MK metabolism is regulated by related genes, its production is exclusively related to strain growth. The effect of sodium selenite on strain biomass resulted in slower metabolism and lower MK content. When the amino acid content of selenium increased, as did the permeability of the cell membrane, the expression levels of genes important for MK synthesis (mokA, mokI, and Lae A) increased [39]. This study revealed that *M. purpureus* undergoes dynamic alterations to adapt to its surroundings. The generation of secondary metabolites increased as the organic selenium level in the culture medium and cells increased, resulting in a rapid increase in the MK content in 6–11 days. After 15 days of culture, the MK concentration in several experimental groups was lower than the detection limit. This may be because MK biosynthetic genes are mutated in selenium-containing media to adjust to the growth environment, resulting in their deactivation.

### 3.6. Biological Activity of Se-Rich Monascus purpureus

*M. purpureus* was cultured for five days under the optimal conditions in the above test and then added to a new culture medium (without adding sodium selenite) for 15 days.

**Figure 11:**
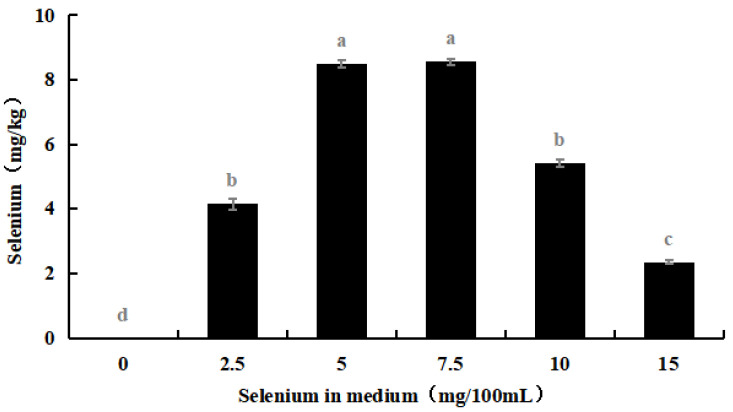
Selenium concentration in secondary culture of Se-rich *M. purpureus.* (The grey lowercase letters in the graph are used for significance difference analysis).

Through simple diffusion, sodium selenite enters *M. purpureus* cells and is converted into protein or polysaccharide selenium, which is then utilized by cells via amino acid or polysaccharide absorption processes. Some selenium ions are absorbed via a sodium pump and hydrogen ion exchange mechanism for exchange and use. As shown in Figure 11, when the selenium concentration in the culture medium increased, the selenium content in the secondary culture of selenium-enriched *M. purpureus* first increased and then decreased.

As shown in Table 3, the consumption rate of selenium by Se-rich *M. purpureus* remained relatively low. The utilization rate of selenium by Se-rich *M. purpureus* reached its maximum (22.99%) at 7.5 mg/100 mL of selenium. During the experiment, Se-rich *M. purpureus* converted a small portion of ingested selenium into selenoproteins, selenopolysaccharides, and other metabolites, which were then transferred to the spores. Selenium fixation is a phase in the process of exporting overloaded cells [40].

*M. purpureus* is frequently utilized in industrial production as a fermentation strain for Monascus rice or Monascus pigments. To better understand the effect of sodium selenite on the growth of *M. purpureus*, a secondary culture of Se-rich *M. purpureus* was inoculated on rice at 8% of the inoculation amount and cultured for 15 days under optimal conditions, and Monascus rice physicochemical indices were determined [41].

As shown in Table 4, the amount of inoculum and the state of the culture medium determined the strength of fungal growth and speed of substrate metabolism during the solid-state fermentation process. At the same time, selenium metabolism and elimination required energy, implying that the high-density fermentation of *M. purpureus* is closely related to the physical and chemical characteristics of Monascus rice; however, the control mechanism of selenium utilization by *M. purpureus* remains unknown.

## 4. Conclusions

In this study, an innovative approach for converting inorganic selenium to organic selenium was devised. Sodium selenite was converted into organic selenium, with SeMet as the major form, along with traces of SeCys2 and MeSeCys, using a functional fermentation strain of *M. purpureus*. The ideal culture conditions for Se-rich *M. purpureus* obtained from the experiment include a selenium content in the culture medium of 7.5 mg/100 mL, a pH of 6.8, a culture temperature of 30 °C, and a rotation speed of 180 rpm. During the selenium-rich culture of *M. purpureus*, the maximum selenium concentration in the cells was approximately 239.17 mg/kg. The composition of the secondary metabolites of Se-rich *M. purpureus* was comparable to that of ordinary cultures. The organic Se concentration in Se-rich *M. purpureus* was 93.45%, the yield of monacolin K was 70.264 mg/L, and the secondary utilization rate of exogenous Se was 22.99%.

The ability of *M. purpureus* to convert different amounts of sodium selenite and the influence of selenium on *M. purpureus*’s growth and metabolism were investigated. Determining more detailed molecular mechanisms requires further research, such as exploring the biological function and effectiveness of Se-rich *M. purpureus* in vivo through mouse experiments to expand the pathways for converting inorganic selenium into organic selenium.

## Figures and Tables

**Table 1:** Experimental factors and coding levels in response surface design.

Horizontal	Factor		
	A pH	B Rotate Speed (r/min)	C Temperature (°C)
−1	6	130	27
0	7	180	30
1	8	230	33

**Table 2:** Response surface design experiment variance analysis and regression coefficient significance test.

Source of Variation	Degree of Freedom	Square Sum	Mean Square	F	P	Statistical Significance
A-pH	1	1.13	1.13	28,013.83	<0.0001	***
B-Temperature	1	0.00784	0.00784	195.23	0.867	
C-speed	1	2.94	2.94	73,150.72	<0.0001	***
A^2	1	14.56	14.56	362,600	<0.0001	***
B^2	1	0.36	0.36	8891.59	<0.0001	***
C^2	1	11.74	11.74	292,400	<0.0001	***
Residual	7	0.000281	0.00004			
Misfit item	9	27.95	3.11	77,337.39	0.5505	
Sum	16	27.95				

Note: In the statistical significance column, *** means extremely significant (*p* < 0.01).

**Table 3:** Selenium utilization of Se-rich *M. purpureus*.

Selenium Addition (mg/100 mL)	0	2.5	5	7.5	10	15
Selenium conversion rate (%)	0	22.93 ± 0.021	22.85 ± 0.014	22.20 ± 0.019	21.51 ± 0.020	21.11 ± 0.021

**Table 4:** Evaluation of Se-rich red koji rice.

Project		Graininess	Powder
Moisture content/%		5.14 ± 0.07	3.48 ± 0.64
Aflatoxin B_1_/(μg/kg)		1.96 ± 0.14	1.66 ± 0.21
Color valence/(μ/g)		3.64 × 10^3^	3.81 × 10^3^
Fineness 150 μm Pass rate/%		-	97.64 ± 0.16
Total arsenic(As)/(mg/kg)		0.16 ± 0.01	0.14 ± 0.01
Heavy metal(Pb)/(mg/kg)		0.038 ± 0.004	0.038 ± 0.005
Microbiological indicators	*Escherichia coli*/(MPN/g)	1.06 ± 0.02	1.05 ± 0.03
	Salmonella/(MPN/g)	-	-
	Shigella/(MPN/g)	-	-
	Staphylococcus aureus/(MPN/g)	-	-

## Data Availability

The data presented in this study are available on request from the corresponding author.
